# Synergistic Effects of Mild Heating and Dielectric Barrier Discharge Plasma on the Reduction of *Bacillus Cereus* in Red Pepper Powder

**DOI:** 10.3390/foods9020171

**Published:** 2020-02-11

**Authors:** Eun Bi Jeon, Man-Seok Choi, Ji Yoon Kim, Shin Young Park

**Affiliations:** Department of Seafood and Aquaculture Science/Institute of Marine Industry, Gyeongsang National University, Tongyeong 53064, Korea; eunb61@naver.com (E.B.J.); pyn358@naver.com (M.-S.C.); yooonn22@naver.com (J.Y.K.)

**Keywords:** *Bacillus cereus*, mild heating, dielectric barrier discharge plasma, red pepper powder, food safety, quality

## Abstract

The synergistic efficacy of a combined treatment of mild heat (MH) and dielectric barrier discharge (DBD) plasma in *Bacillus cereus*-contaminated red pepper powder was tested. A cocktail of three strains of *B. cereus* (NCCP 10623, NCCP 14579, ATCC 11778) was inoculated onto red pepper powder and then treated with MH (60 °C for 5–20 min) and DBD plasma (5–20 min). Treatment with MH and DBD plasma alone for 5–20 min resulted in reductions of 0.23–1.43 and 0.12–0.96 log CFU/g, respectively. Combined treatment with MH and DBD plasma was the most effective at reducing *B. cereus* counts on red pepper powder, and resulted in log-reductions of ≥6.0 log CFU/g. The largest synergistic values (4.24–4.42 log) against *B. cereus* in red pepper powder were obtained by the combination of 20 min MH and 5–15 min DBD plasma. The values of Hunter color ‘‘L’’, ‘‘a’’, and ‘‘b’’, were not significantly different from those of the untreated sample and that with the combination of MH (60 °C for 5–20 min) and DBD plasma (5–20 min). Also, no significant (*p* > 0.05) differences in pH values between samples were observed. Therefore, these results suggest that the combination of MH treatment and DBD plasma can be potentially utilized in the food industry to effectively inactivate *B. cereus* without incurring quality deterioration of red pepper powder.

## 1. Introduction

Red pepper powder is an essential dietary spice in Korea, and the principle ingredient and determinant of the color, taste, and quality of traditional fermented *kochujang* (fermented red pepper paste). This spice is also commonly used as a cooking sauce for processed foods (e.g., Korean ramen noodles, as well as traditional Korean soups and stews) [1]. Because the cultivation period of red peppers occurs during hot and humid seasons, they are likely to be contaminated by pests or microorganisms. Moreover, there is a high likelihood of microbial cross-contamination during red pepper powder processing, the extent of which depends on the sanitary conditions of the operator, manufacturing facility, and surrounding environment. Harvesting red peppers and drying them in a conventional manner (i.e., with a dryer, using sunlight) includes no sterilization process, leaving them exposed to potential contamination from many microorganisms [2]. The use of contaminated red pepper powder in a food can lead to rapid spoilage. In a study by Chun et al. [3], the contamination level in red pepper powder was 6.72 and 6.57 log CFU/g for aerobic bacteria and *Bacillus cereus*, respectively.

Plasma is a quasi-neutral ionized gas state composed of ions, free electrons, atoms, and molecules in their fundamental or excited states with a net neutral charge [4]. Plasma technology is commonly used in biomedical industries, semiconductor manufacturing, and displays (e.g., fluorescents, televisions, monitors, lighting, and equipment) [5]. Plasma can be artificially generated by subjecting a neutral gas to a wide range of temperatures and pressures; therefore, it is classified into thermal and non-thermal plasma (NTP). NTP, also named cold plasma, is a promising new food sterilization technology, and is proven to be effective against pathogenic microorganisms with relatively little impact on the nutritional value of a treated food. Dielectric barrier discharge (DBD) plasma is used as an NTP, and can be generated between two electrodes that are covered by polylactic acid [6]. DBD plasma is a type of discharge suitable for food processing because it can be applied to a large area [7]. Kim et al. [8] reported that microwave-powered cold plasma at 900 W and 667 Pa for 20 min inhibited naturally occurring total aerobic bacteria in red pepper powder by approximately 1 log CFU/g.

Pasteurization, a relatively mild heat (MH) treatment in which food is heated to <100 °C, is commonly used in the food industry. It is also frequently applied as a critical control point in the hazard analysis critical control point (HACCP) system. Hurdle technology (a combination of non-thermal and thermal processing techniques) can control the growth of spoilage and pathogenic microorganisms in foods, thus extending the shelf-life of foods [9]. UV-C irradiation/hot air heating or cold plasma treatment/hot water immersion have been shown to specifically inactivate foodborne pathogens in red pepper powders [8,10]

To date, a few studies have researched combinations of non-thermal and thermal treatments on reducing foodborne pathogens in pepper powders. Cheon et al. [10] reported that the combination of ultraviolet radiation with MH treatment was more effective than UV-C irradiation alone for inactivation of *Escherichia coli* and *Salmonella*. Moreover, this combined approach does not cause quality deterioration of powdered red pepper. Choi et al. [11] reported that microbiological analysis of powders inoculated with *E*. *coli* O157:H7 and *Staphylococcus aureus*, following a radio frequency thermal treatment and plasma treatment combination, resulted in 2 log CFU/g reductions in the microbial counts proportional with increasing treatment cycles. 

There is a need to examine the potential synergistic effects of combined treatment with MH and DBD plasma for the decontamination of red pepper powder. The present study was therefore undertaken to determine the synergistic effects of a combined treatment with MH (60 °C for 5–20 min) and DBD plasma (5–20 min) on the reduction of *B. cereus* in red pepper powder.

## 2. Materials and Methods

### 2.1. Bacterial Strain

Three strains of *B. cereus* (NCCP 10623, NCCP 14579, ATCC 11778) were tested. A stock culture (10^8^ CFU/mL) was maintained at −80 °C in tryptic soy broth (TSB; Difco Laboratories, Detroit, MI, United States) containing 30% glycerol. Bacteria were cultured on tryptic soy agar (TSA; Difco Laboratories, Detroit, MI, United States) plates at 37 °C for 24 h. Afterwards, a single colony was inoculated into TSB and grown at 37 °C for 12 h with shaking at 150 *× g*.

### 2.2. Culture Preparation

Each strain was cultured in 5 mL of TSB at 37 °C for 24 h, harvested by centrifugation at 5400 *× g* for 10 min at 4 °C, and washed three times with 0.85% sterile saline water. The final pellets were resuspended in 10 mL of 0.85% sterile saline water, corresponding to approximately 10^7^–10^8^ CFU/mL. Suspended pellets from each of the three *B. cereus* strains were combined into a mixed culture cocktail at a final concentration of approximately 10^7^ CFU/mL for use in this study.

### 2.3. Inoculation and Sample Preparation

Commercially available dried red pepper powders were purchased at a traditional market (Tongyeong, Korea). Red pepper powder samples were autoclaved for 15 min at 121 °C before inoculation to remove preexisting microorganisms. For inoculation, 1 mL of the culture cocktail was added to 25 g of samples in 500 mL glass beakers, and then mixed with a sterilized stainless-steel spoon for 5 min to ensure uniform distribution. After mixing, samples were dried for 1 h inside a clean bench (25 ± 1 °C) with the fan running. The concentration of final samples was 10^6^–10^7^ CFU/g.

### 2.4. Mild Heat Treatment and Dielectric Barrier Discharge Plasma Treatment

To pasteurize using MH treatment, the sterilized petri dish containing 25 g of red pepper powder was sealed with parafilm (Heathrow Scientific, Vernon Hills, IL, United States) to prevent water vapors from going into the sample. Samples were treated at 60 °C in a water bath for 5, 10, 15, and 20 min, and then immediately cooled in an ice water bath for 5 min to stop further thermal inactivation. 

**Figure 1:**
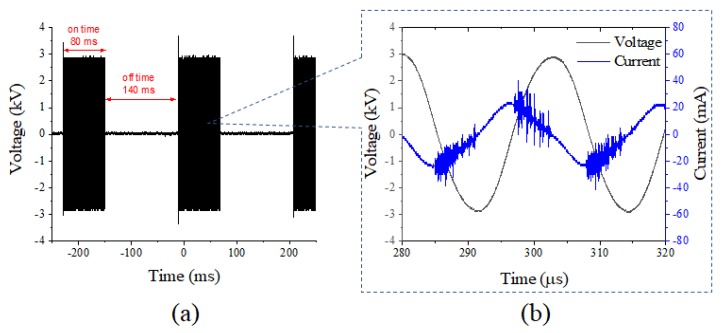
(**a**) voltage waveform with on-time and off-time duty; (**b**) voltage (black line) and discharge current (blue line) curve versus time.

**Figure 2:**
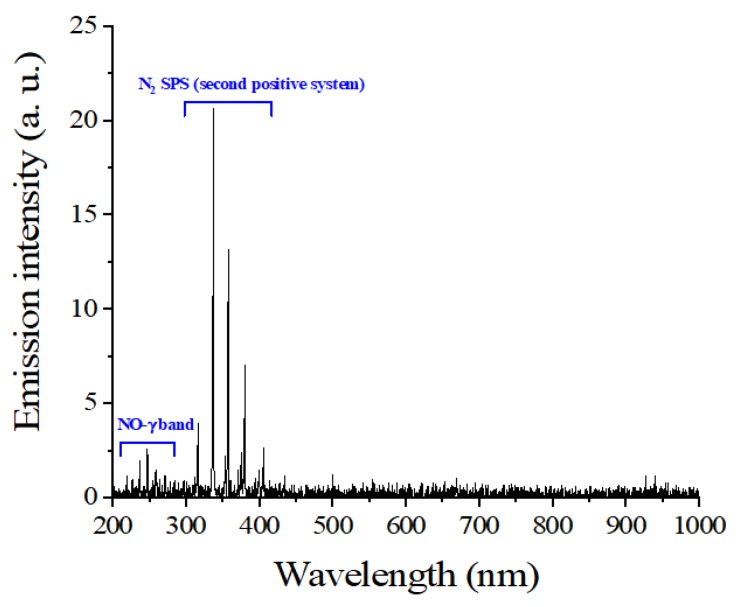
Optical emission profile of dielectric barrier discharge (DBD) plasma.

Fiveplicate samples of 5 g of red pepper powder were treated with DBD plasma. The DBD plasma device (μ-DBD Surface Plasma Generator, Model; Micro DBD plasma) was supplied by Plasma Biomedicine Institute (Plasma Bioscience Research Center, Seoul, Korea) and was described by Ryu et al. [7]. The silver electrode which served as a high voltage electrode was screen printed (thickness of 10 µm) on glass (thickness of 1.8 mm); the dielectric material (consisting of SiO_2_) was also screen printed to 100 µm in polylactic acid. A metal mesh grid was attached on the rear side of the glass and used as a grounded electrode. Gas flow can be guided to the mesh surface by a polylactic acid cover via a gas injection hole. DBD plasma was generated on the rear glass surface between the glass and metal mesh grid using a nitrogen flow rate of 1.5 L per minute. The DBD plasma, under a driving frequency 43 kHz, showed voltage and current characteristics with low discharge voltage of approximately 1 kV and discharge peak current 40 mA, respectively. The minimum discharge voltage for plasma production by DBD plasma devices used in this experiment was 1.1 kV. The optical emission profile was measured 3 mm from the mesh surface with a 10 s integration time, using a spectrometer (HR4000, Ocean optics). Figure 1 shows the typical DBD current and voltage characteristics that first discharge peak current 40 mA occurs at discharge voltage 1 kV, under driving frequency 43 kHz and applied voltage 3 kV. The optical emission profile has been measured at 2 mm away from the mesh surface with 10 s integration time using a spectrometer (HR4000, Ocean optics). Figure 2 shows the NO- γ band emission (200–300 nm) and N_2_ SPS (second positive system) emission (300–400 nm) with the wavelength. The device was turned on at least 10 min before the start of the experiment, and the surface of the red pepper powder previously inoculated with *B. cereus* was treated with DBD plasma for 5, 10, 15, and 20 min in a sterile petri dish (35 × 15 mm). A distance of 3 mm was maintained between the plasma-emitting electrode and the sample during treatment. 

### 2.5. Determining Synergistic Reduction Effects

To estimate any synergistic effect on bacterial inactivation, the inactivation values of combined MH and DBD plasma were compared with those of MH or DBD plasma alone. The combination was conducted by applying MH as a first disinfectant and DBD plasma as a second disinfectant, because MH may have weakened or destroyed the cell membranes of bacteria, causing them to be more susceptible to the new physical disinfectants, such as DBD plasma. The disinfection efficacy after different treatments was determined by measuring microbial reduction. The synergistic reduction effect values of combined MH and DBD plasma were calculated using the following equation:

(1) Value of synergistic reduction=A−(B+C)

where *A* is the reduction from combined mild heating and DBD plasma disinfection, *B* is the reduction from MH treatment disinfection alone, and *C* is the reduction from DBD plasma disinfection alone. On the basis of this equation, synergistic and antagonistic reduction effects are indicated as plus values and minus values, respectively.

### 2.6. Microbial Enumeration

For the enumeration of *B. cereus*, a treated red powder sample (25 g) was transferred into a sterile stomacher bag (Labplas Inc., Sainte-Julie, Quebec, Canada) containing 225 mL of 0.85% sterile saline water, and then homogenized in a stomacher (EASY MIX, AES Chemunex, Rennes, France) for 2 min based on Korean Food Code [12]. After homogenization, sample aliquots were serially diluted tenfold in 9 mL blanks of 0.85% sterile saline water, and 0.1 mL of diluted samples were duplicate spread-plated onto selective media. TSA was used as a media for the enumeration of *B. cereus*. All plates were incubated at 37 °C for 48 h before counting.

### 2.7. Quality Measurement

In order to characterize any potential changes in the quality of red pepper powder after combined treatment with MH and DBD, color and pH factors were assessed. All treated samples were stored in a clean bench for 1 h to allow for equilibration to room temperature. Subsamples of powdered red peppers were selected from three random locations.

#### 2.7.1. Hunter Color

After treatment with MH and DBD plasma, the colors of red pepper powders were measured using a color difference meter (UltraScan PRO, Hunterlab, United States), which was calibrated with the original value from a standard plate (“L” = 98.48, “a” = 0.14, and “b” = 0.41). “L” (brightness+, darkness–), “a” (redness+, greenness–), and “b” (yellowness+, blueness–) values were measured, and the mean of the three measurements was recorded for each sample.

#### 2.7.2. pH Values

To measure pH, 100 mL of distilled water was added to 1 g of red pepper powder, stirred at room temperature for 5 min, and filtered using Whatman paper (Whatman inc., Piscataway, NJ, United States). The supernatant was measured three times using a pH meter (A211, Thermo Orion, Benchtop, MI, United States).

### 2.8. Statistical Analysis

Data are presented as the mean of three determinations ± standard deviation. One-way analysis of variance (ANOVA) was performed using the SPSS software system. Bacteria enumeration presented as log CFU/g reductions (treated vs. pretreated), color, and pH values were analyzed with the Duncan’s multiple range test to identify any potential differences among mean values. Statistical significance was tested at the 5% probability level (*p* < 0.05).

## 3. Results

### 3.1. Synergistic Reductions of Bacillus cereus in Red Pepper Powder were Achieved by Combined Treatment with Mild Heat Treatment and Dielectric Barrier Discharge Plasma

To characterize the combined treatment effect of MH and DBD plasma on *B. cereus*-contaminated red pepper powder, reductions in *B. cereus* counts were determined (Table 1). After 5, 10, 15, and 20 min of treatment with MH alone, *B. cereus* counts were significantly (*p* < 0.05) reduced—by 0.23, 0.93, 1.16, and 1.43 log CFU/g, respectively. *B. cereus* counts were also significantly (*p* < 0.05) reduced by 0.12, 0.28, 0.61, and 0.96 log CFU/g after treatment with DBD plasma alone for 5, 10, 15, and 20 min, respectively. Treatment with DBD plasma resulted in a decrease of 0.96 log CFU/g after the 20 min maximum treatment, while MH treatment for 10 min resulted in a decrease of 0.93 log CFU/g. These results indicate that *B. cereus* reduction was primarily dependent on MH treatment rather than DBD plasma. 

After combined treatment with MH and DBD plasma, *B. cereus* counts in contaminated red pepper powder were reduced by 0.33–6.43 log CFU/g. The initial concentration of *B. cereus* inoculated on red pepper powder was measured as 7.14 ± 0.10 log CFU/g. This combined treatment led to decreases of more than 1 log CFU/g in most combinations, except for 5 min MH + 5 min DBD plasma (0.33 log CFU/g), and 5 min MH + 10 min DBD plasma (0.42 log CFU/g). As the MH treatment time and DBD plasma treatment time increased, the reduction effect was greater. Synergistic effects were greater among these combination treatments (20 min MH + 10 min DBD plasma, 20 min MH + 15 min DBD plasma, and 20 min MH + 20 min DBD plasma). Also, the maximum reduction of *B. cereus* was 6.43 log CFU/g after treatment with a combination of 20 min MH and 20 min DBD plasma. The results of these experiments demonstrate that DBD plasma used in our standard conditions led to a greater reduction in *B. cereus* compared with each individual treatment. 

Table 2 reveals the synergistic effects of MH treatment combined with DBD plasma on the reduction of *B. cereus* in contaminated red pepper powder. Among them, synergistic effects were observed for most combination treatments, except for 5 min MH + 5 min, 5 min MH + 10 min DBD plasma, 10 min MH + 5 min DBD plasma, and 10 min MH + 10 min DBD plasma. Unfortunately, antagonistic effects did appear in certain combinations, including 5 min MH + 5 min DBD plasma (–0.22 reduction) and 5 min MH + 10 min DBD plasm (–0.28 reduction). Additionally, the maximum synergistic reduction values of greater than 4 log CFU/g (99.99%) were achieved when samples were treated with MH for 20 min and DBD plasma for 5 min, 10 min, or 15 min. 

### 3.2. Hunter Color and pH Value on red Pepper Powder Treated by Combined Mild Heat Treatment and Dielectric Barrier Discharge Plasma

To identify any potential mechanical color differences between combined treatments, Hunter color ‘‘L’’ (lightness), ‘‘a’’ (redness), and ‘‘b’’ (yellowness) values were measured on the red pepper powder (Table 3). Hunter color ‘‘L’’, ‘‘a’’, and ‘‘b’’ values of the combination treated samples were not significantly (*p* > 0.05) different from those of non-treated samples. Also, there were no significant (*p* > 0.05) differences in pH values among the treated samples (Table 4).

## 4. Discussion

*B. cereus* is a soil bacterium [13] and is commonly found in raw plants, including rice, potatoes, peas, beans, herbs, and spices [14]. Additionally, *B. cereus* is regarded as a food poisoning bacterium that can occasionally be an opportunistic human pathogen [15]. *B. cereus* can survive harsh environments, including normal cooking temperatures. Herbs and spices are the main source of spore-forming bacteria like *Bacillus* spp. And *Clostridium* spp. in food products (e.g., soups, cooked or stewed dishes, sauces), which can provide good conditions for the growth of these microorganisms. *B. cereus* has also been shown to cause food poisoning in consumers [16]. It was also reported that *B. cereus* was considered as the main pathogenic microorganism detected in red pepper powder [3,17,18]. Red pepper powder is of agricultural origin, and is therefore generally contaminated by this bacterium during the cultivation, drying, grinding, and storage processes.

Various methods, such as UV irradiation, chlorine dioxide treatment, infrared treatment, radiation, and electron beam irradiation, have been used to reduce microorganism contamination of red pepper powder [19,20]. Although these methods have proven effective at removing pathogenic bacteria, they may also lead to undesirable chemical (lipid oxidation) and sensory (color, odor and texture) changes in seafoods and vegetables [21]. Based on these studies, the current study used a combination of MH and DBD plasma to sterilize red pepper powder, in the hopes that it would not affect quality or leave any chemical residues. 

Many studies on the inactivation of pathogenic bacteria in vegetables by MH treatment have been conducted. Son et al. [22] reported that spinach inoculated with *E. coli* Ol57:H7 decreased from 5.98 log CFU/g to 4.16 log CFU/g after treatment at 60 °C for 5 min (1.82 log CFU/g decrease). The results of this study are in line with the findings of Koseki et al. [23]. They reported that lettuce treated at 50 °C reduced the surviving numbers of each *E. coli* O157:H7 and *Salmonella* by 2.73 and 2.81 log CFU/g, respectively. Such MH treatments are also capable of inactivating microbial spores and many enzymes and toxins present in foods. Generally, MH treatment can kill microbes by altering their cell membranes and denaturing proteins [24]. These results indicate that water washing without mild heat is insufficient to inactivate microbes.

In the food processing industry, cold plasma treatment represents an innovative technology, especially since it has been proven to be effective against foodborne pathogens with relatively little effect on food nutritional value [25]. Yong et al. [26] reported that the populations of *E. coli*, *S.* Typhimurium, and *L. monocytogenes* on cheese slices treated with plasma for 5 min (approximately 5 log CFU/g) were decreased by 1.75, 1.97, and 1.65 log CFU/g, respectively. Deng et al. [27] observed that DBD treatment for 30 s at 16 kV, 20 kV, and 25 kV could yield 1-, 2.43-, and 4.12-log reduction of *E. coli* counts on almond samples. This decrease is high compared with the present results; however, it is considered to be caused by differences in the voltage and frequency of the plasma. Similar to the results presented here, Won et al. [28] determined that the growth of *E.coli* on onion powders inoculated with *E. coli* O157:H7 treated with atmospheric plasma for 5, 10, 15, and 20 min were inhibited by 0.4, 0.8, 0.9, and 1.4 log CFU/g, respectively. We may speculate that compared to our study, the reductions in *E. coli* O157:H7 was greatly influenced by food type; as the moisture in food increased, the efficiency of the plasma against bacteria on the food increased. Muhammad et al. [29] observed that when tiger nut milk was treated with DBD plasma for 8 min, aerobic bacteria and fungi were reduced to 0.97 log CFU/g and 0.94 log CFU/g, respectively. Bauer et al. [30] noted that when DBD plasma was directly applied to the surface of vacuum-packaged beef loin contaminated with *S. aureus* for 1 min, the counts of this bacteria were reduced by roughly 1.7 log CFU/g.

There are a variety of active species in the activated plasma (e.g., electrons, cations, anions, free radicals, ultraviolet photons). Cold plasma for the inactivation of microorganisms is mainly associated with the reactive species generated, particularly reactive nitrogen species (RNS) and reactive oxygen species (ROS) [31]. Rupturing bacterial cell walls using a build-up of charged particles or bombardment with free radicals have been proposed as possible modes of action for bacteria inactivation [31]. Although greater microbial inhibition is achieved by increasing the non-thermal treatment power and time, in practice a possible negative effect on quality must be taken into account [8,26]. For these reasons, there has been increasing interest throughout the world in employing hurdle approaches to enhance the shelf life of red pepper powder and to ensure the microbiological safety of the products in food production and processing. Gayán et al. [32] studied the synergistic inactivation of *E. coli* and *Salmonella enterica* when treated with UV-C light at mild temperature. Simultaneously treatment with sanitizers, UV-irradiation and MH was shown to be more effective than either treatment alone or when applied sequentially. Ha and Ha [33] reported that combined sanitizers (ethanol, hydrogen peroxide, and sodium hypochlorite)/UV treatments resulted in greater reductions in bacterial counts compared with either treatment alone. Xiang et al. [34] reported that *E. coli* O157:H7 was reduced from about 8.28 log CFU/mL to undetectable levels following exposure to the combined treatment of plasma-activated water and 60 °C (approximately 8 log CFU/mL reduction). Kim et al. [35] reported that red pepper flakes inoculated with *B. cereus* spores decreased from 6.0 log CFU/g to 3.4 log CFU/g after exposure to microwave-combined cold plasma for 20 min (2.60 log CFU/g decrease). Seong et al. [36] reported that *E. coli* O157:H7, *S.* Typhimurium, and *L. monocytogenes* on fresh lettuce were reduced by 1.55, 2.39, and 2.45 log CFU/g, respectively, following combined treatment with cold plasma and UV-C. The combinations of MH treatment and DBD plasma with other food preservation technologies not only allow the optimization of microbial inactivation, but also effectively maintain the nutritional and sensory attributes of food products during storage [37]. 

To evaluate the suitability of this combined decontamination approach, quality properties of red pepper powder were measured. The results from the Hunter color and pH value assessments demonstrated the potential for combined treatment with MH and DBD plasma, as bacterial decontamination approach did not noticeably alter the color properties of red pepper powder. According to Li et al. [38], here was no significant difference between the treatments as a result of examining the surface color, texture, and overall acceptability of potato with ascorbic acid and mild heat treatment. In general, due to the non-thermal nature, cold plasma treatments have shown no or minimal impacts on the physical, chemical, nutritional, and sensory attributes of various products [39]. However, Pankaj et al. [4] noted that reactions of RNS and ROS with food components can produce undesirable volatile compounds and carcinogens, destroy essential nutrients, and change the functionalities of proteins, lipids, and carbohydrates. Therefore, the concentrations of RNS and ROS generated by the plasma production will be measured in our coming future studies. Moreover, it will be investigated whether RNS and ROS may affect essential and functional nutrients, and to which extent these radicals may also have an impact on food quality in the studies. 

Combining treatment with MH and DBD plasma is more effective at inactivating *B. cereus* compared with MH treatment alone. Also, in our antagonism results (−0.22 ± 0.6 and −0.28 ± 0.7), the average results are shown to be antagonistic. However, because of the wide range of standard deviations, it is difficult to determine accurately by either antagonism or synergism. Moreover, this combined approach does not lead to a deterioration in the quality of red pepper powder. Thus, this combined treatment can be utilized as an alternative to conventional decontaminating interventions (e.g., super-heated steam). 

## 5. Conclusions

The current combined MH and DBD plasma is cost-effective (e.g., easy-to-handle, no food quality changes) compared to other possible treatment processes. It was specifically noted here that a combined treatment of red pepper powder with MH and DBD plasma was more effective compared with treatment with MH or DBD alone. *B. cereus* counts in contaminated red pepper powder were significantly (*p* < 0.05) reduced as treatment times with MH and DBD plasma increased. Reduction values for combined treatment with MH and DBD plasma against *B. cereus* in red pepper powder were 0.12 to 6.43 log CFU/mL, respectively. The largest synergistic reduction values (4.24–4.42 log CFU/mL) of *B. cereus* in red pepper powder were obtained by a combination of 20 min MH and 5~15 min DBD plasma. In addition, it appears that the degree of microbial reduction can be affected by many factors (e.g., plasma type, food type, or matrix, such as moisturized food and dried food, food acidity, treatment duration time, and type of bacteria). Hunter color ‘‘L’’, ‘‘a’’, and ‘‘b’’ values of the combination-treated samples were not significantly (*p* > 0.05) different from those of non-treated samples. Also, there were no significant (*p* > 0.05) differences in pH value. This combined treatment approach did not result in any deterioration in the quality of red pepper powder. The results of this study reveal that the reductions of >6-log CFU for *B. cereus* in red pepper powder were accomplished by a combination of 20 min MH and 5–15 min DBD plasma. These results suggest that combined treatment with MH and DBD plasma is a potentially a novel method to improve the microbial safety of dried spices, including red pepper powder.

## Figures and Tables

**Table 1:** Reduction of *Bacillus cereus* in red pepper powder after the combined treatment of MH and DBD plasma.

	Mean (± SD) Reduction Value (log CFU/g)					
	DBD Plasma Treatment (min)					
Target Organism	MH Treatment (min)	0	5	10	15	20
*B. cereus*	0	–	0.12 ± 0.21	0.28 ± 0.32	0.61 ± 0.30	0.96 ± 0.17
	5	0.23 ± 0.24	0.33 ± 0.20	0.42 ± 0.10	1.97 ± 0.26^gh^	2.57 ± 0.33^f^
	10	0.93 ± 0.18	1.45 ± 0.23^ij^	1.70 ± 0.13^hi^	3.08 ± 0.32^e^	3.64 ± 0.24^d^
	15	1.16 ± 0.18^j^	2.34 ± 0.19^fg^	2.60 ± 0.24^f^	3.87 ± 0.22^d^	5.53 ± 0.16^c^
	20	1.43 ± 0.23^ij^	5.77 ± 0.26^bc^	6.05 ± 0.27^ab^	6.20 ± 0.31^ab^	6.43 ± 0.21^a^

The data indicates means with standard deviations (SDs) (three samples/treatment). MH: mild heat. DBD plasma: dielectric barrier discharge plasma. Gray box: Among all combination treatments having >1 log reduction, means with different letters (a~j) differ significantly (*p* < 0.05) by the Duncan multiple range test.

**Table 2:** Synergistic and antagonistic effects of *B*. *cereus* in red pepper powder after the combined treatment with MH and DBD plasma.

	Mean (± SD) Synergistic and Antagonistic Value of Reduction (log CFU/g)				
	DBD Plasma (min)				
Target Organism	MH Treatment (min)	5	10	15	20
*B. cereus*	5	–0.22 ± 0.60	–0.28 ± 0.71	1.24 ± 0.32^d^	1.20 ± 0.65^d^
	10	0.47 ± 0.30	0.57 ± 0.91	1.65 ± 0.61^cd^	1.58 ± 0.51^cd^
	15	1.13 ± 0.42^d^	1.24 ± 0.13^d^	2.18 ± 0.33^c^	3.20 ± 0.64^b^
	20	4.32 ± 0.51^a^	4.42 ± 0.32^a^	4.24 ± 0.72^a^	3.83 ± 0.43^ab^

The data indicates means with standard deviations (three samples/treatment). MH: mild heat. DBD plasma: dielectric barrier discharge plasma. Synergistic effects indicated as + = (reduction achieved by the combination of MH + DBD plasma) – ((reduction achieved by MH) + (reduction achieved by DBD plasma)). Antagonistic effects indicated as – = (reduction achieved by the combination of MH + DBD plasma) – ((reduction achieved by MH) + (reduction achieved by DBD plasma)). Gray box: Among all combination treatments having >1 log synergistic reductions, means with different letters (a~d) differ significantly (*p* < 0.05) by the Duncan multiple range test.

**Table 3:** Effects of combined treatment of MH and DBD plasma on the Hunter color of red pepper powder.

	Hunter color		
Treatment	“L” value	“a” value	“b” value
Control	26.88 ± 0.17	22.01 ± 0.23	14.12 ± 0.23
5 min MH + 5 min DBD	26.76 ± 0.21	22.08 ± 0.13	14.09 ± 0.25
5 min MH + 10 min DBD	26.65 ± 0.18	22.15 ± 0.24	14.07 ± 0.20
5 min MH + 15 min DBD	26.60 ± 0.10	22.07 ± 0.11	14.13 ± 0.14
5 min MH + 20 min DBD	26.66 ± 0.14	22.09 ± 0.16	14.17 ± 0.18
10 min MH + 5 min DBD	26.72 ± 0.18	22.12 ± 0.21	14.21 ± 0.11
10 min MH + 10 min DBD	26.61 ± 0.12	22.16 ± 0.26	14.16 ± 0.18
10 min MH + 15 min DBD	26.63 ± 0.14	22.12 ± 0.18	14.12 ± 0.08
10 min MH + 20 min DBD	26.64 ± 0.07	22.06 ± 0.14	14.21 ± 0.15
15 min MH + 5 min DBD	26.65 ± 0.28	22.08 ± 0.21	14.24 ± 0.14
15 min MH + 10 min DBD	26.76 ± 0.24	22.06 ± 0.24	14.13 ± 0.16
15 min MH + 15 min DBD	26.64 ± 0.15	22.12 ± 0.14	14.21 ± 0.19
15 min MH + 20 min DBD	26.77 ± 0.16	22.16 ± 0.16	14.09 ± 0.21
20 min MH + 5 min DBD	26.75 ± 0.22	22.01 ± 0.19	14.18 ± 0.18
20 min MH + 10 min DBD	26.76 ± 0.18	22.06 ± 0.12	14.11 ± 0.17
20 min MH + 15 min DBD	26.64 ± 0.13	22.12 ± 0.16	14.22 ± 0.17
20 min MH + 20 min DBD	26.77 ± 0.23	22.16 ± 0.14	14.09 ± 0.14

The data indicates means with standard deviations (three samples/treatment). Control: non-treated sample. Values with the same letter in the same column are not significantly different (*p* < 0.05) by Duncan’s multiple range test. Hunter “L” values = lightness; Hunter “a” values = redness+, greenness–; Hunter “b” values = yellowness+, blueness−.

**Table 4:** Effects of combined treatment of MH and DBD plasma in the pH value of red pepper powder.

	Mean (±SD) Reduction Value (log CFU/g)					
	DBD Plasma (min)					
	MH Treatment (min)	0	5	10	15	20
pH	0	4.86 ± 0.04	4.89 ± 0.02	4.87 ± 0.03	4.88 ± 0.03	4.84 ± 0.02
	5	4.87 ± 0.04	4.90 ± 0.02	4.88 ± 0.03	4.86 ± 0.04	4.84 ± 0.03
	10	4.85 ± 0.03	4.79 ± 0.02	4.87 ± 0.03	4.86 ± 0.03	4.75 ± 0.03
	15	4.84 ± 0.05	4.80 ± 0.03	4.81 ± 0.04	4.77 ± 0.04	4.76 ± 0.05
	20	4.83 ± 0.05	4.80 ± 0.04	4.78 ± 0.05	4.74 ± 0.05	4.73 ± 0.04

The data indicates means with standard deviations (three samples/treatment).
